# Serological and molecular surveillance of leishmaniosis in dogs across the United States

**DOI:** 10.1186/s13071-026-07589-6

**Published:** 2026-08-01

**Authors:** Daniel Felipe Barrantes Murillo, Rachel C. Smith, K. Wade Burton, Chengming Wang, Theresa Wood, Subarna Barua, Guilherme G. Verocai, Maureen A. Kelly, Pablo D. Jimenez Castro, Christian M. Leutenegger, Jeremiah T. Saliki, Kathryn T. Duncan

**Affiliations:** 1 Department of Veterinary Pathobiology, College of Veterinary Medicine, Oklahoma State University, Stillwater, OK USA; 2 Oklahoma Animal Disease Diagnostic Laboratory, Oklahoma State University, Stillwater, OK USA; 3 IDEXX Laboratories, Inc., Westbrook, ME USA; 4 Department of Pathobiology, College of Veterinary Medicine, Auburn University, Auburn, AL USA; 5 Department of Veterinary Pathobiology, College of Veterinary Medicine and Biomedical Sciences, Texas A&M University, College Station, TX USA; 6 Antech Diagnostics, Loveland, CO USA

**Keywords:** Canine leishmaniosis, *Leishmania infantum*, SNAP *Leishmania* Test, Vector-borne disease PCR panel, IFA, Sand fly, Digital PCR, Real-time quantitative PCR

## Abstract

**Background:**

Canine leishmaniosis (CanL) is caused by *Leishmania* species. While vertical transmission is the primary route of CanL transmission in the USA, the role of sand fly vectors in its transmission and the prevalence in the broader pet population remain under-investigated. Diagnosis of CanL may include serology, PCR, or histopathology. However, ELISA tests are seldom used for the diagnosis of CanL in the USA despite their preference in endemic regions.

**Methods:**

Firstly, a serological and molecular survey was conducted in Oklahoma and Texas using the SNAP™ *Leishmania* Test (IDEXX Laboratories Inc.) on canine blood or serum, while serum only was used for commercial indirect immunofluorescence assay (IFA) testing. Molecular testing for *Leishmania* spp. included targeting segments of the small subunit rRNA gene by qPCR, the minicircle kinetoplast using a dPCR assay (QIAGEN), and a confirmatory Vector-borne Disease Panel (Antech Diagnostics). Secondly, sand fly trapping and testing were conducted in Oklahoma and Texas with morphological and molecular identification targeting the *ITS-2* gene, as well as qPCR for *Leishmania* spp. Finally, a nationwide serological survey of apparently healthy dogs was conducted using the SNAP *Leishmania* Test and confirmatory testing with a commercial IFA test.

**Results:**

In the regional survey, seroprevalence was 0.61% (2/328; CI 0.07–2.0) with the SNAP *Leishmania* Test and 4.35% (6/138; CI 2.0–9.0) via the IFA test. *Leishmania* spp. DNA was not detected in any of the tested blood samples (*n* = 253). *Psathyromyia shannoni* (*n* = 8) were collected from Denton and Grayson Counties, Texas, with no detection of *Leishmania* spp. DNA. The nationwide prevalence of *Leishmania* spp. antibodies was 0.35% (9/2,515; CI 0.16–0.68) in non-Foxhound pet dogs from 48 states. Notably, all positive samples on the SNAP *Leishmania* Test were positive on IFA testing.

**Conclusions:**

*Leishmania* seropositive animals were found in Texas and Oklahoma, as other infections were demonstrated by the vector-borne panel. The sand fly vector role could not be determined due to limited collection of specimens. Nationwide survey demonstrated a low prevalence of *Leishmania* antibodies. The methods used in this study (SNAP *Leishmania* Test and IFA) constitute useful tools for canine leishmaniosis surveys, and sample choices should be taken into account.

**Graphical Abstract:**

**Figure:**
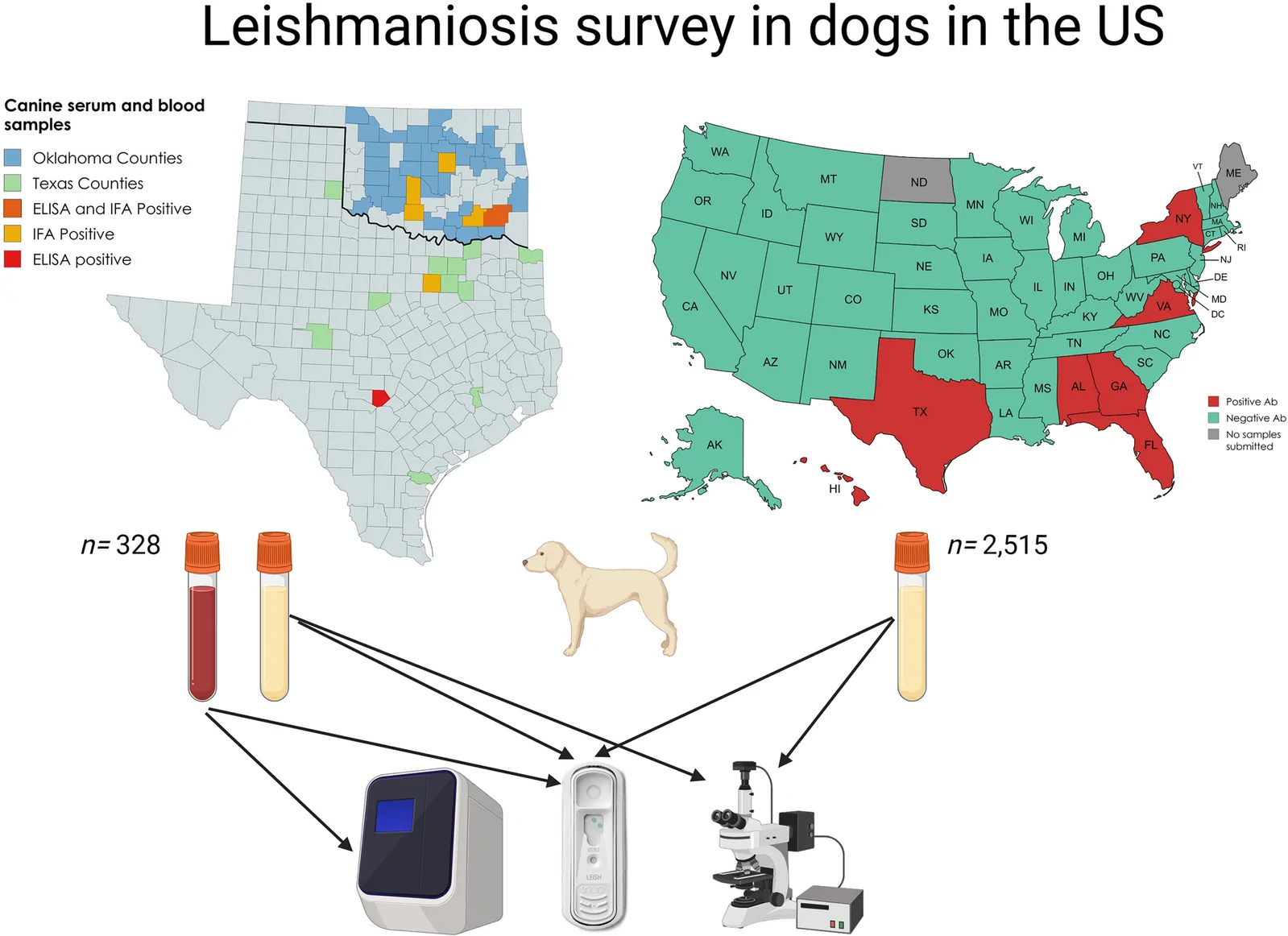

**Supplementary Information:**

The online version contains supplementary material available at https://doi.org/10.1186/s13071-026-07589-6.

## Background

Canine leishmaniosis (CanL) is a potentially fatal disease in dogs, caused by parasites from the genus *Leishmania* (Kinetoplastida: Trypanosomatidae) and transmitted by phlebotomine sand flies (Diptera: Psychodidae: Phlebotominae) [1]. The specific epidemiological features of CanL in the USA have been previously described, but re-evaluation is necessary due to continued biogeographical changes and resultant habitat modifications for the primary vectors [2]. Additionally, domestic dog travel and importation for adoption purposes have continued to grow both nationally and internationally, leading to the emergence or re-emergence of many significant vector-borne pathogens [3, 4]. It is known that *Leishmania infantum* is the most important cause of CanL in the USA [5]. This parasite is widely distributed in North America, with a higher prevalence in foxhound dogs and other hunting breeds [5–8]. Notably, vertical transmission appears to be the primary mode of transmission of CanL in the USA within these groups [5, 7, 9–13]. *Lutzomyia longipalpis* can become infected after feeding on apparently healthy dogs vertically infected with *L. infantum* [14]*.* Therefore, these dogs should be considered as a potential reservoir source of *L. infantum*, as the disease is more commonly diagnosed or spreads to these non-endemic regions in the USA [14, 15]. In the USA, *Dampfomyia anthophora* (syn. *Lutzomyia anthophora*), *Psathyromyia shannoni* (syn. *Lutzomyia shannoni*), and *Lutzomyia diabolica* are known vectors of *Leishmania* species [15]. *Dampfomyia anthophora* and *Lutzomyia diabolica* can transmit *Leishmania mexicana* to hamsters in laboratory conditions [16, 17]. *Psathyromyia shannoni* can experimentally transmit *Leishmania infantum* in dogs [18]. Of these, *P. shannoni* is extensively distributed throughout the southeastern USA, elevating concern for the transmission risk of CanL in this region [19–22].

The diagnosis of CanL can be achieved by the following methods used alone or preferably in combination: (1) detection of specific serum antibodies (IgG) through semiquantitative diagnostic techniques like indirect fluorescent antibody (IFA) or enzyme-linked immunosorbent assay (ELISA); (2) qualitative rapid tests as commercial ELISA tests and rapid immunochromatographic tests (ICT); (3) detection of *Leishmania* spp. genomic DNA by conventional polymerase chain reaction (PCR) or quantitative real-time PCR (qPCR) targeting *Leishmania*-specific genes; (4) or the visualization of *Leishmania* spp. amastigotes by cytology or histopathology in fluids or tissues [23, 24]. Previous epidemiological surveys on *Leishmania* spp. in the USA relied on serological and molecular methods, specifically IFA testing and/or PCR [6, 8, 10, 25–27]. ELISA kits commercially available in other countries have not been available for use in *Leishmania* spp. surveillance in canine patients in the USA. Given that the most recently published epidemiological survey in the USA was conducted between 2018 and 2019 [25, 27], the current study aims to update and describe in more detail the current epidemiology of CanL in high-risk populations. To optimize sampling efforts, a detailed epidemiologic regional survey was first conducted on canine samples and targeted sand fly trapping in Oklahoma and Texas, where the risk of infection is high, given past reports and sand fly suitability [5, 19, 22]. Following this preliminary study, a national seroprevalence survey was conducted on 2,515 convenience samples from dogs to provide information for veterinarians and public health officials outside a high-risk area, which may trigger additional investigations.

## Methods

### Regional canine sampling and testing

#### Canine blood and serum samples

A total of 328 convenience dog blood and/or serum samples from Texas and Oklahoma originally submitted to the Oklahoma Animal Disease Diagnostic Laboratory (OADDL) between March 2024 and March 2025 were retrieved. Breed, sex, age, and geographical location (state and county) from each case were collected. The identification of the samples included a distinction between owned dogs and shelter dogs. An additional 109 whole blood samples collected from shelter dogs in Kendall County, Texas, were included. No foxhounds were included in this regional survey.

#### Serological testing

Each blood or serum sample (*n* = 328) was tested using a commercial ELISA, the Canine *Leishmania* Antibody Test Kit (SNAP™ *Leishmania* Test*,* Product Code 5047.U1; IDEXX Laboratories, Inc., Westbrook, Maine, USA), following the manufacturer's instructions. This test is not currently approved by the FDA for commercial use in the USA; thus, test samples were obtained and imported strictly for research use under USDA authorization (ML 281281).

Testing via IFA was done with canine serum samples from Oklahoma (*n* = 111) and Texas (*n* = 27) using the commercial *Leishmania* IFA Canine IgG Antibody Kit (Catalog No. LPD-120, Fuller Laboratories, Fullerton, California, USA). Briefly, the serum was tested in a semiquantitative assay using a single dilution (1:40); 25 µL of serum was diluted in 975 µL of 1X Phosphate-Buffered Saline (PBS), and 10 µL of the dilution was transferred to each slide well. The assay included positive and negative controls in two separate wells. The slides were incubated in a humidity chamber or a CO_2_ incubator for 30 min, at 37° ± 0.5 °C, at 50% relative humidity. Then, the slides were gently rinsed four times with PBS, without allowing these to dry, and, in a dark room, 10 µL of the affinity-purified DyLight 488-labeled rabbit anti-canine IgG (heavy chain, Fuller Laboratories, Fullerton, California, USA) with bovine serum albumin and Evans’ blue counterstain was added. The slides were returned to the humidity chamber for 30 min of incubation at 37° ± 0.5 °C in a dark room. After the incubation, the slides were gently washed four more times before applying four drops of mineral oil and covered with a coverslip to visualize under a fluorescence microscope (Nikon Eclipse TS100) at 40× magnification. A positive reaction appeared as bright, sharp, and regular staining of the promastigote outer membrane; patterns of reactivity different than that seen in the positive control were considered non-specific and negative.

#### Molecular testing

Molecular testing was conducted on blood samples from Oklahoma (*n* = 96) and Texas (*n* = 157), using three different assays after DNA extraction. Briefly, 200 µL of blood was extracted using the QIAamp DNA Blood Mini Kit (QIAGEN, Germantown, Maryland, USA) using the QIAcube Connect instrument according to the manufacturer's instructions. First, a validated qPCR was used to target the partial small subunit rRNA gene of *Leishmania* spp. to obtain a 60-bp amplicon. The assay utilized the primers LEIS.U1 (5′-AAGTGCTTTCCCATCGCAACT-3′), LEIS.L1 (5′-GACGCACTAAACCCCTCCAA-3′), and the probe LEIS.P1 (5′-[6-FAM]-CGGTTCGGTGTGTGGCGCC-MGBNFQ-3′) as previously described [28]. The TaqMan (20x) working solution contained the primers LEIS.U1 (900 nM), LEIS.L1 (900 nM), and the probe LEIS.P1 (250 nM). Each PCR reaction contained 10 µL master mix (TaqMan™ Fast Advanced Master Mix, Catalog No. 4444558, Thermo Fisher Scientific, Waltham, Massachusetts, USA), 1 µL TaqMan (20X) working solution, 7 µL nuclease-free water, and 2 µL DNA template. The PCR was performed using the Quant Studio™ 3 Flex Real-Time PCR System (Thermo Fisher Scientific) with the following thermocycling conditions: polymerase activation 60 s at 95 °C, 45 cycles of denaturation 3 s at 95 °C, and annealing/extending 30 s at 60 °C. The positive control was a gBlocks™ Gene Fragments synthetic dsDNA standard (Integrated DNA Technologies, Coralville, Iowa, USA) of the target sequence at a dilution containing 50 copies/1 μL of target DNA.

For confirmatory purposes, extracted DNA from each sample were submitted for *Leishmania* spp. PCR targeting the minicircle kinetoplast partial gene as part of the commercially available vector-borne disease PCR panel from Antech Diagnostics, Inc., Mars Petcare Science & Diagnostics. This panel also included qPCR tests for the detection of the following infectious agents: *Mycoplasma haemocanis, Trypanosoma cruzi, Anaplasma platys, Bartonella clarridgeiae/quintana, Hepatozoon canis/felis, Anaplasma phagocytophilum, Ehrlichia canis, Ehrlichia ewingii, Ehrlichia chaffeensis, Babesia* spp. (incl. Coco), *Babesia gibsoni, Babesia conradae, Babesia canis, Cytauxzoon felis, Babesia gibsoni/Cytauxzoon felis* Atovaquone Resistance Marker, *Bartonella henselae, Hepatozoon americanum,* Spotted Fever Group *Rickettsia (Rickettsia* spp.*), Neorickettsia risticii, Candidatus Mycoplasma haemominutum/haematoparvum, Candidatus Mycoplasma turicensis,* and *Borrelia* spp., and an internal sample control (ISC) to quantify the nucleic acid for quality control purposes. The qPCR was run in 12 μL qPCR reactions using 5 μL DNA extract in a LightCycler 480 (Roche Diagnostics, Inc., Indianapolis, IN) using the manufacturer’s recommended thermos-cycling protocols.

The digital polymerase chain reaction (dPCR) screening was performed using the QIAcuityDx Universal MasterMix Kit (QIAGEN) on a QIAcuity One 5plex Instrument. The Microbial DNA dPCR assay targeted the minicircle kinetoplast gene from *Leishmania* spp. (Catalog No. DMA00771, QIAGEN). Each reaction had a final volume of 40 µL, containing 10 µL of master mix, 2 µL of 20× primer–probe mix solution, 24.0 µL of molecular-grade water, and 4 µL of DNA template. The following thermocycling conditions were used: polymerase activation 2 min at 95 °C, followed by 40 cycles of denaturation 15 s at 95 °C and annealing/extension 60 s at 58 °C. The positive control was a gBlocks™ synthetic dsDNA standard (Integrated DNA Technologies, Coralville, Iowa, USA) of the target sequence at a dilution containing 50 copies/1μL of target DNA.

### Regional sand fly sampling and testing

#### Collection sites and sand fly trapping

From April to August 2025, eight farms were visited at least once for sand fly collection, for a total of 24 collection nights. Farms were in Payne (Farm 1), Logan (Farm 2), Creek (Farm 3), and Rogers (Farm 4) counties in Oklahoma and Johnson (Farm 5), Grayson (Farms 6 and 8), and Denton (Farm 7) counties in Texas. Oklahoma and Texas were targeted for collections due to previous reports of sand fly collection within McCurtain County, Oklahoma [29], and Bexar, Caldwell, Collin, Medina, and Lamar Counties in Texas [30–32]. Additionally, 14 endemic cases of human cutaneous leishmaniasis caused by *Leishmania* spp. or *Leishmania mexicana* have been reported in southeastern Oklahoma [29] as well as northeastern (Caldwell, Collin, Dallas, Denton, Ellis, Hill, Lamar, Tarrant Counties) [29, 32, 33] and southcentral Texas (Washington County) [34]. The time of the year was chosen to avoid months with a mean monthly temperature below 10 °C, given that sand flies are unlikely to be present under those conditions [31].

Following approval from landowners, sand flies were trapped using the UVA-LED Light Trap (John W. Hock Company, Gainesville, Florida, USA) placed on farms with domestic animals (i.e., dogs, cats, horses, cows, poultry) cohabiting with humans near forests. Three UVA-LED Light traps were placed on each property and distributed across: (1) domestic sites (abandoned buildings, cracks in mud floors and walls); (2) peridomestic sites (animal burrows, chicken coops, earth dikes, soil cracks, wells, dry excreta from domestic animals, under stones); and (3) sylvatic sites (dry cesspits, drains, bird nests, soils at base of the trees, leaf litter on forest floor, under rocks, small animal burrows, terrestrial nests, roots of large trees and caves) [35]. The traps were 30 to 50 m apart to avoid interference and were located in areas with natural light, avoiding artificial light, fumes, or smoke. GPS coordinates were recorded for each sampling site using an iPhone 14 Plus. The traps were suspended 1 to 1.5 m from the ground and turned on at dusk (around 6:00 pm CDT) until dawn (6:00 am CDT) for 12 trapping-hours per collection night. Traps were set for three consecutive nights and were avoided during periods of heavy, continuous rain. Additionally, 0.4 to 1 kg of dry ice was placed above each trap as bait. At the end of the collection period, the sampling bag was placed in a cooler for 2 to 5 min at − 20 °C to kill the collected insects before transferring to a petri dish.

#### Sand fly morphological and molecular identification

The head and the last three tergite segments of the sand flies were dissected and mounted in Permount® mounting medium (Fischer Scientific, Waltham, Massachusetts, USA) to identify, through light microscopy, key taxonomic anatomical structures [22, 35]. The remaining portion of the body was used for genomic DNA extraction using the DNeasy Blood & Tissue Kit (QIAGEN), in the QIAcube Connect System following the manufacturer’s instructions. To maximize DNA yield, the sand flies were incubated in a 1.5-mL microcentrifuge tube containing 20 µL of proteinase K (QIAGEN) and 180 µL of ALT Buffer (QIAGEN) at 56 °C for 90 min and shaken at 2000 rpm.

For the molecular identification of the sand flies, a qPCR targeting the internal transcribed spacer 2 (*ITS-2*) gene to obtain a 235-bp amplicon using the previously described primers: SF-ITS2F (5′-ACTGCAGGACACATGAACATC-3′) and SF-ITS2Rr (5′-CCTGGTTAGTTTCTTTTCCTCC-3′) [36]. The qPCR was performed using the PowerTrack™ SYBR Green Master Mix for qPCR (Thermo Fisher Scientific) in the Quant Studio 3 Flex Real-Time PCR System (Thermo Fisher Scientific). Each reaction had a final volume of 20 µL, containing 10 µL of master mix, 0.8 µL of SF-ITS2F primer (400 nM), 0.8 µL of SF-ITS2Rr primer (400 nM), 4.15 µL of molecular-grade water, and 4.25 µL of DNA template. The thermocycling conditions were as follows: enzyme activation for 2 min at 95 °C, followed by 40 cycles of denaturation for 15 s at 95 °C, annealing/extending for 1 min at 58.8 °C, and a dissociation step for 15 s at 95 °C, 1 min at 58.8 °C, and 15 s at 95 °C. Amplicons were purified using the QIAquick PCR Purification Kit (QIAGEN) and sent for bidirectional Sanger sequencing at the Oklahoma State Molecular Core Facility (Stillwater, Oklahoma, USA). Following assembly of consensus sequences with Geneious Prime® (version 2024.0.4, GraphPad Software), sequence identity was determined by BLASTN comparison against published sequences in the National Center for Biotechnology Information (NCBI) database, GenBank.

#### *Detection of Leishmania *spp.* in collected sand flies*

*Leishmania* spp. detection in all the sand flies collected was performed by qPCR targeting the small subunit rRNA partial gene to obtain an amplicon size of 60 bp, which utilized the primers LEIS.U1, LEIS.L1, and the probe LEIS.P1 as described above [28].

### National canine sampling and testing

Convenience serum samples from apparently healthy pet dogs were retrieved between January 2023 and July 2024 from the Serology Laboratory at Auburn University (Auburn, Alabama, USA). The samples were originally submitted for rabies antibody testing (Fluorescent Antibody Virus Neutralization, FAVN, or Rapid Fluorescent Focus Inhibition Test, RFFIT), primarily for the purpose of domestic or international pet transportation. Before rabies antibody testing, the samples were incubated at 56 °C for 30 min and stored at − 20 °C after being used. The previous incubation step does not cause significant denaturation of IgG antibodies, since denaturation requires higher temperatures and longer incubation [37, 38]. Basic demographic data were obtained including sex, breed, age, and the state of origin. Samples were assigned to geographical regions, including Northeast, Southeast, Midwest, and West, according to previously defined regional divisions [39]. No Foxhounds were included in this nationwide survey.

Each sample (*n* = 2,515) was tested using the SNAP *Leishmania* Test kit (IDEXX Laboratories Inc.) according to the manufacturer’s instructions. The tests were obtained and used under USDA authorization (ML 286621). To match current standards available in the USA, positive samples were confirmed using the *Leishmania* IFA Canine IgG Antibody Kit (Fuller Laboratories) as previously described.

## Results

### Serological and molecular prevalence of *Leishmania* spp. in dogs from Oklahoma and Texas

**Fig. 1:**
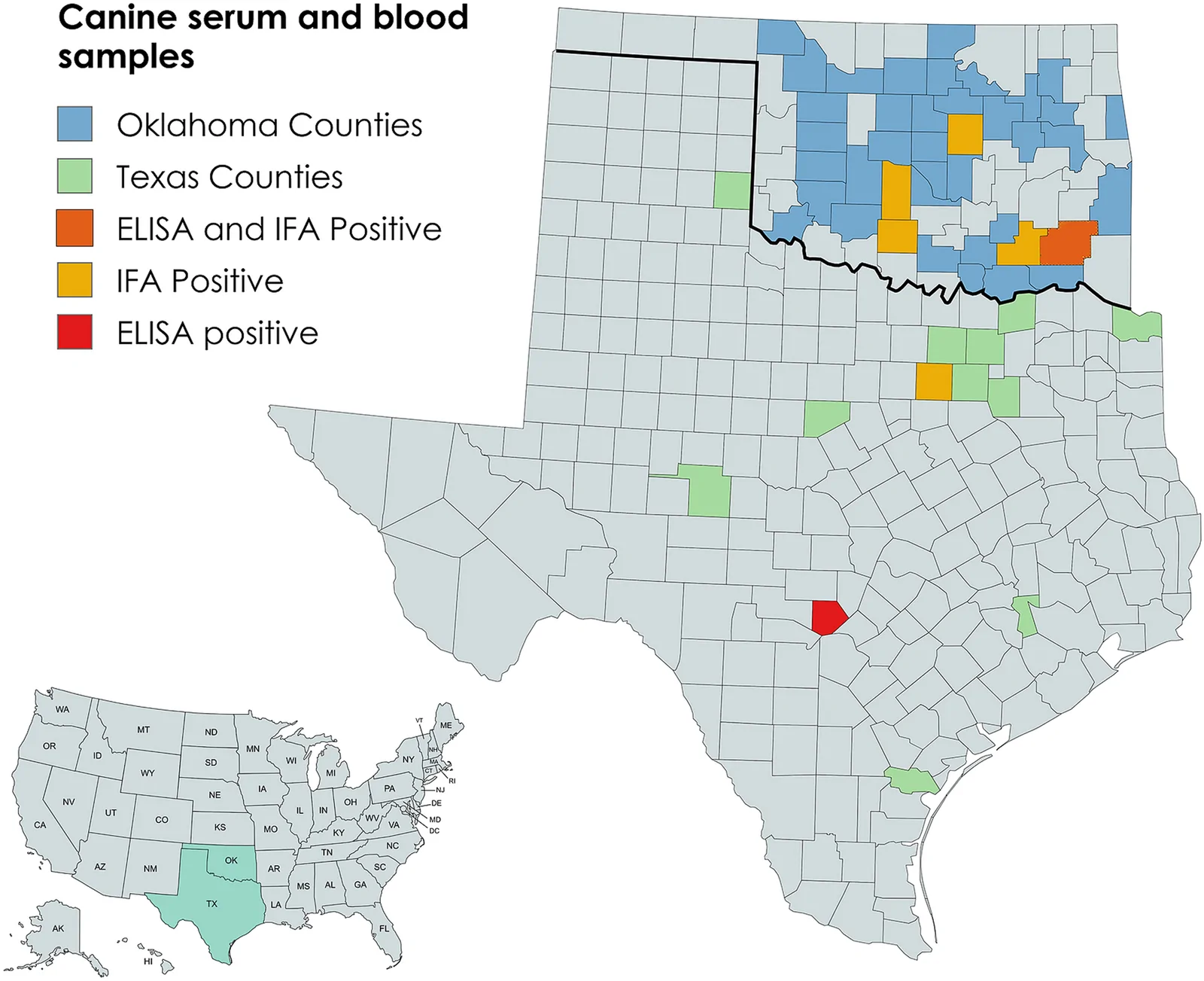
Geographical distribution of blood and serum samples from dogs from Oklahoma and Texas used in a serological *Leishmania* sp. survey testing by ELISA (SNAP™ *Leishmania* Test) and IFA test. The positive sample on the ELISA (Kendall County, Texas) could not be tested on the IFA because only blood was available

A total of 328 dog samples were collected from Oklahoma (*n* = 161) and Texas (*n* = 167). SNAP™ *Leishmania* Test *****was performed on all samples, whereas IFA testing was performed on only a subset of 138 serum samples, due to sample restrictions for this assay. PCR testing for detection of *Leishmania* spp. was performed on a subset of 253 samples (Fig. 1, Additional file 1: Data Set S1). Overall prevalence determined by the SNAP *Leishmania* Test was 0.6% (2/328) [CI 0.07–2.0], which included one dog from a shelter in Kendall County, Texas, and one dog from Pushmataha County, Oklahoma (Table 1, Additional file 1: Data Set S1). Prevalence determined by IFA was 4.3% (6/138) [CI 2.0–9.0], which included one dog from Tarrant County, Texas, and five dogs from Oklahoma (Atoka, Grady, Lincoln, Stephens, and Pushmataha counties) (Table 1, Additional file 1: Data Set S1). The dog from Pushmataha County, Oklahoma was positive on both the ELISA and IFA tests. No *Leishmania* spp. DNA was detected by any of the PCR assays (qPCR or dPCR) performed. Because a comprehensive vector-borne disease panel (Antech Diagnostics, Loveland, CO) was performed, additional infections detected included *M. haemocanis* 1.2% (3/255) [CI 0.2–3.0], *T. cruzi* 0.8% (2/253) [CI 0.01–3.0], *A. platys* 0.4% (1/253) [CI 0.01–2.0], *B. clarridgeiae/quintana* 0.4% (1/253) [CI 0.01–2.0], and *H. canis* 0.4% (1/253) [CI 0.01–2.0] (Additional file 1: Data Set S1). Both dogs with detectable *T. cruzi* were located at the shelter in Kendall County, Texas, and were seronegative for *Leishmania* spp. on ELISA (SNAP *Leishmania* Test) and IFA testing.

**Table 1:** Positive percentage, and [95% confidence interval] results for *Leishmania* spp. testing in dogs from Oklahoma and Texas

State	ELISA positive percentage	IFA positive percentage	PCR positive percentage
Oklahoma	0.6% (1/161) [CI 0.02–3.00]	3.7% (1/27) [CI 0.02–4.31]	0% (0/96) [CI 0.00–0.03]
Texas	0.6% (1/167) [CI 0.02–3.00]	4.5% (5/111) [CI 1.00–10.00]	0% (0/157) [CI 0.00–0.02]
Total	0.6% (2/328) [CI 0.07–2.00]	4.3% (6/138) [CI 2.00–9.00]	0% (0/253) [CI 0.00–0.01]

### Morphological and molecular identification of collected sand flies

**Fig. 2:**
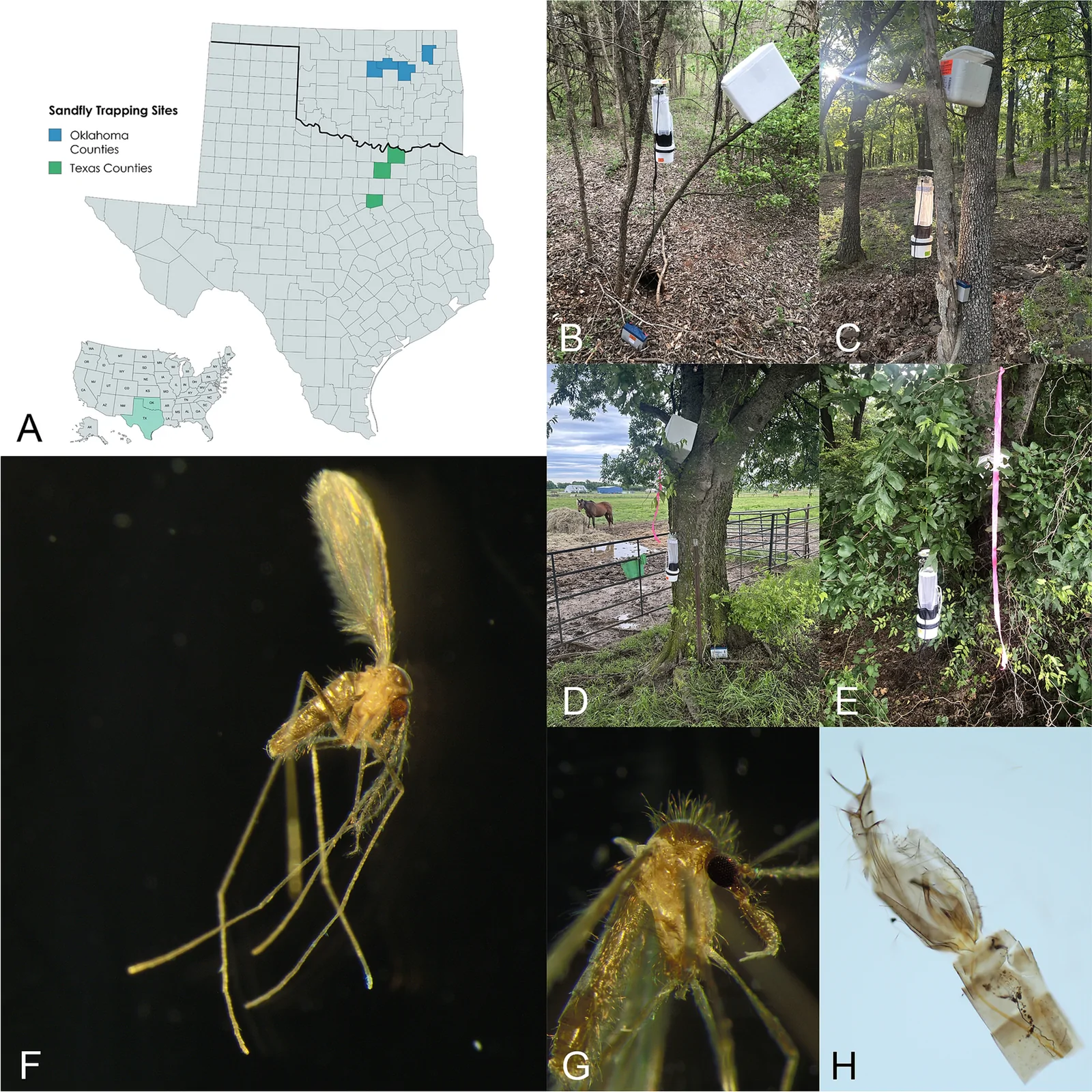
Details and results of regional sand fly trapping and collection with examples of preferred sand fly habitats (**B**–**E**). **A** Geographical distribution of the Oklahoma and Texas collection sites. **B** UVA-LED Light Trap placed in Logan County, Oklahoma near an animal burrow. **C** UVA-LED Light Trap placed in Creek County, Oklahoma over leaf litter on the forest floor. **D** UVA-LED Light Trap placed in Johnson County, Texas, near muddy soil and rotted manure. **E** UVA-LED Light Trap placed in Grayson County, Texas, over soil at the base of a tree. **F** Female *Psathyromyia shannoni* trapped in Grayson County, Texas, with long legs and a slender body. **G** Female *Psathyromyia shannoni* collected in Grayson County, Texas, with a hairy appearance and a short proboscis. **H** Mounted aedeagus from a male *Psathyromyia shannoni* collected in Grayson County, Texas. Gonostylus has four strong spines, and the lateral lobe is shorter than the gonocoxite

Eight sand flies were collected (seven females and one male) from Denton and Grayson Counties, Texas (Fig. 2). All specimens were morphologically and molecularly identified as *P. shannoni* (Additional file 2: Data Set S2)*. Leishmania* spp. DNA was not detected in any of the collected sand flies by qPCR.

### Nationwide seroprevalence of *Leishmania* in dogs from the USA

**Fig. 3:**
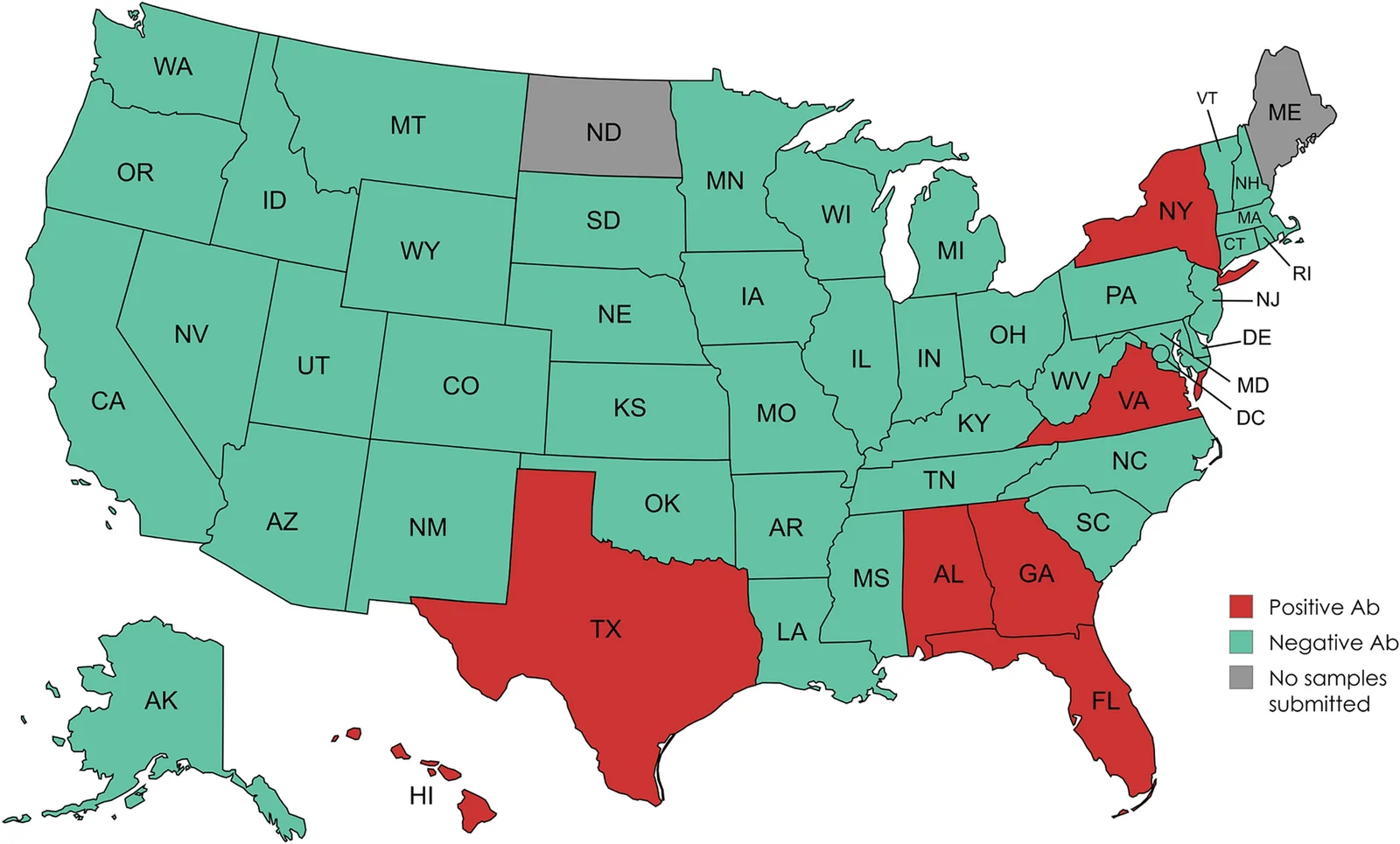
Geographic origin of dogs with ELISA-positive samples on SNAP™ *Leishmania* Test in the USA

A total of 2,515 canine serum samples were collected from 48 states, plus the District of Columbia, and 0.35% (9/2,515) [CI 0.2–0.7] of the samples tested positive for *Leishmania infantum* antibodies using the SNAP *Leishmania* Test (Additional file 3: Data Set S3). Positive animals were identified in three geographical regions: Southeast [Alabama (*n* = 1), Florida (*n* = 2), Georgia (*n* = 1), Texas (*n* = 1), Virginia (*n* = 1)], Northeast [New York (*n* = 2)], and West [Hawaii (*n* = 1)] (Table 2, Fig. 3). Dogs with positive results ranged from 2–12 years old, eight were males, [Chihuahua (*n* = 1), Yorkshire Terrier (*n* = 2), Border Collie (*n* = 2), mixed breed dogs (*n* = 3), and not specified (*n* = 1)] and 1 female (Chihuahua *n* = 1). All samples positive by the SNAP™ *Leishmania* Test were concurrently positive for *Leishmania* antibodies on the IFA test.

**Table 2:** National ELISA results of canine seroprevalence survey for *Leishmania infantum*

Region	State (no. positive)	Number tested	Positive percentage	95% Confidence interval
Northeast	New York (*n* = 2)	528	0.38	[0.01–1.5]
Southeast	Alabama (*n* = 1), Florida (*n* = 2), Georgia (*n* = 1), Texas (*n* = 1), Virginia (*n* = 1)	1,105	0.54	[0.02–1.2]
Midwest	0	150	0	[0.00–3.0]
West	Hawaii (*n* = 1)	732	0.14	[0.00–0.9]
Total positives	9	2,515	0.35	[0.2–0.7]

## Discussion

The wide distribution of *Leishmania* in dogs has been previously described in surveys across the USA [7, 8, 27], including Texas [25, 26, 40], but not Oklahoma. Previous studies using serological or molecular methods determined a higher prevalence of *Leishmania* spp. In dogs from the USA, contrasting with the findings of this study. However, such surveys were performed on populations considered high-risk. Reported *Leishmania* spp. Prevalence ranges from 8.9% to 41.0% in surveys performed on foxhounds and other hunting breeds [6–8], and 2% to 14.3% in military animals [25, 40]. The most recent survey on dogs utilizing IFA or PCR diagnostics determined a prevalence of 6.4%, including foxhounds and non-foxhound breeds, with 86.9% of the positive dogs having a travel history from endemic areas for leishmaniosis outside of the USA [26]. A study conducted using dog samples initially submitted for tick-borne disease diagnosis, between 2000 and 2001, determined an overall prevalence of 0.2% by IFA testing in non-foxhound dogs [27]. Thus, the findings of this current project align with this previous survey, concluding that the prevalence of *Leishmania* spp. is apparently low in non-foxhound canine populations in the USA.

Serological tests are widely used for the diagnosis of CanL, as other techniques that require demonstrating the presence of amastigotes are more time-consuming, invasive, expensive, and potentially less sensitive [41]. Seroconversion in infected animals requires at least 3 months, with circulating antibodies against *Leishmania* spp. persisting at high titers for approximately 2 years [41]. To date, ELISA testing has not been used in *Leishmania* spp. surveys in the USA [6–8, 25, 26, 40], partially due to the non-availability of commercial tests. In general, the SNAP™ assays provide a convenient, easy-to-use device capable of performing an ELISA for antigen or antibody detection with timely, in-clinic results [42]. In Brazil, a study evaluated the previous version of the commercial ELISA, the SNAP™ CLATK Canine *Leishmania* Antibody Test Kit, Product Code 5047.U0 (IDEXX Laboratories Inc., Westbrook, ME, USA) determining a sensitivity and specificity of 94.7% (95% CI 91.41–97.01%) and 90.6% (95% CI 83.80–95.21%), respectively, with a high degree of agreement (*k* = 0.8445) between results of the SNAP™ test, IFA, and cytology [41]. Most of the false positives occurred with samples seropositive for *T. cruzi,* since both agents are members of the Trypanosomatidae family with similar surface antigens [41]. Cross-reactions between *L. infantum* and *Leishmania chagasi* were wrongfully documented [41], since *L. infantum* and *L. chagasi* are widely considered the same species [43]; this cross-reaction cannot occur, and cross-reactions among other *Leishmania* species were not reported. These limitations are not currently reported for the new SNAP *Leishmania* Test used in this project, which is specifically designed for the detection of *L. infantum* [44]. In fact, in this survey, *T. cruzi* was detected by PCR from the blood of two shelter dogs from Kendall County, Texas. Both were negative for *Leishmania* antibodies on ELISA and IFA tests. Since *Leishmania* spp. infection was not molecularly confirmed in this survey by PCR; further investigation for potential cross-reactivity between several *Leishmania* species or other etiologies is warranted. Other infectious agents with reported cross-reactivity on the previous version of the SNAP test were not included (*Toxoplasma gondii, Neospora caninum)* or detected (*Babesia canis,* and *Ehrlichia canis*) through the vector-borne disease diagnostic panel [41].

The SNAP *Leishmania* Test offers several advantages over IFA testing, such as being less time-consuming, requiring less personnel training and specialized instruments, and is applicable with anticoagulated whole blood (heparin or EDTA), plasma or serum [41]. Additionally, IFA testing has the disadvantage of cross-reactivity with other infectious agents, including *T. cruzi, E. canis, T. gondii,* and even *Demodex canis* antibodies [26, 27, 41, 45–48]. The interpretation of results can be subjective or precluded by non-specific fluorescence if the serum is hemolytic or lipemic. For instance, an IFA test can be recorded as “positive” due to the subjective interpretation of the human reader, depending on the specific dilution and methodological differences [49]. Thus, the combination of serological tests (IFA and ELISA) can improve the detection and achieve the research objectives [49]. In this study, more positive samples were detected by IFA than by ELISA (4.3% vs 0.6%). Compared with ELISA, IFA has lower specificity and sensitivity in some studies [36, 40], and IFA has greater variability than ELISA [49]. IFA tests are able to identify *Leishmania-*positive samples; however, false-positive reactions have been observed in the 1:40 to 1:160 dilutions of an in-house IFA test in one study [50]. IFA tests typically increase the false-positive rate in surveys from endemic canine leishmaniosis regions [51]. These IFA limitations align with our results. ELISA has a good sensitivity and specificity with an overall higher number of true positives, whereas the IFA test has a high number of true positives but less agreement with other serological tests [49]. Only one sample from Pushmataha County, Oklahoma, was positive on both ELISA and IFA in the regional survey. This was a 3-month-old Cocker Spaniel; this could be early; however, the seroconversion in naturally infected dogs varies from 1 to 22 months after exposure [52]. The transmission from a sand fly vector seems unlikely in this case, and the results might reflect circulating maternal antibodies through placenta or colostrum that eventually fade away after 3 to 4 months or represent a cross-reaction with other *Leishmania* species like *Leishmania mexicana.* However, it is unknown if the ELISA or IFA used in this study has such cross-reactions since no positive controls were tested with other *Leishmania* species. The other positive sample on the ELISA (Kendall County, Texas, USA) could not be tested on the IFA because only blood was available. All ELISA-positive samples were also positive on IFA in the nationwide survey. Due to budget limitations, IFA testing was not performed in all 2,515 samples from the nationwide survey; however, we speculate that the positive percentage would be higher compared to the commercial ELISA used, as observed in the regional survey. Due to variation in the validation of the commercial IFA assay used (i.e., specificity and sensitivity not specified by the manufacturer), cross-reaction with other infectious agents, non-specific fluorescence, and subjective interpretation of results might have influenced the reported results in this study, but these limitations are inherent in IFA tests.

The molecular survey did not yield positive or detected results for *Leishmania* spp. even though molecular methods were comprehensive, highly sensitive, and included the use of qPCR and dPCR targeting several conserved genes among *Leishmania* species. Digital PCR is a new and emerging molecular diagnostic technology that offers several advantages over PCR and qPCR, making it especially appealing for vector-borne pathogen diagnosis, including *Leishmania* spp. The advantages of dPCR include: absolute quantification of copy numbers, accurate quantification when amplification is low, elimination of the standard curve, and improved accuracy in the presence of inhibitors [53]. The application of dPCR for the detection of *Leishmania* has been seldom explored, but has the potential to overcome the limitations of qPCR with low copy numbers which is particularly helpful when optimal sampling cannot take place (i.e., blood vs bone marrow). Lack of detection of *Leishmania* DNA in this study is unsurprising, since blood samples are considered a less sensitive molecular diagnostic sample in comparison with bone marrow, lymph node, spleen, skin, or conjunctival swabs [23]. Molecular diagnosis, unlike serological tests, can assist in determining an infection and the exact *Leishmania* spp. species responsible for infection [23]. A positive or detected PCR test should be interpreted as a *Leishmania* spp. infection, but not necessarily leishmaniosis without correlation to clinical signs [23].

Several limitations are present in this study and must be disclosed. The SNAP *Leishmania* Test is currently not FDA-approved or commercially available in the USA, and the lack of known positive samples from *L. infantum-*infected dogs in the present study precluded a direct comparison between the two serological assays used. Additionally, because samples (i.e., blood or serum) were collected opportunistically and under non-invasive measures, the negative PCR results should not be used as definitive evidence for the absence of *Leishmania* infection in the Southcentral region of the USA, since blood is less sensitive for the detection of the agent. However, cases of subclinical leishmaniosis cannot be completely ruled out, given that the convenience samples have limited information regarding travel history, and most of the animals are presumed to be apparently healthy. Finally, even though the locations of the captured sand flies aligned with the large geographical distribution reported for *P. shannoni* [5, 19, 22], the low numbers of collected specimens precluded further investigations regarding the potentially relevant distribution in domiciliary, peridomiciliary, and sylvatic environments due to the limited trapping sites and time frame.

## Conclusions

Nationally, CanL seroprevalence is apparently low in the USA; however, the prevalence of antibodies is not restricted to hunting breeds as previously believed. *Leishmania* antibodies are present in dogs from Texas and Oklahoma. The potential role of *P. shannoni* as a vector of CanL in the Southcentral USA could not be determined, and further investigation of the distribution of this dipteran is warranted. The SNAP *Leishmania* Test and IFA tests represent a valuable tool for the detection of circulating antibodies for CanL infection. Further investigation of potential cross-reactions between different *Leishmania* species, including *Leishmania mexicana* and other infectious agents, is warranted where geographically relevant. The molecular detection of *Leishmania,* even with highly sensitive methods like qPCR and dPCR, must not rely on blood samples alone and instead should utilize optimal tissue samples when available.

## Supplementary Information

Additional file 1: Data Set S1. Demographic information, serological, and molecular survey results of the 328 canine samples retrieved from Oklahoma and Texas.

Additional file 2: Data Set S2. Date, location, morphological, and molecular identification of sand flies collected in Oklahoma and Texas.

Additional file 3: Data Set S3. Demographic information and ELISA test results from the nationwide serological survey for *Leishmania *sp. in the USA.

## Data Availability

All data generated or analyzed during this study are included in this published article [and its supplementary information files].

## Abbreviations

CanL: Canine leishmaniosis
dPCR: Digital polymerase chain reaction
ELISA: Enzyme-linked immunosorbent assay
FAVN: Fluorescent antibody virus neutralization
GPS: Global Positioning System
IFA: Indirect fluorescent antibody assay
ITS-2: Internal transcribed spacer 2
OADDL: Oklahoma Animal Disease Diagnostic Laboratory
PBS: Phosphate-buffered saline
RFFIT: Rapid fluorescent focus inhibition test
qPCR: Real-time polymerase chain reaction
